# Effect of long-term methylene blue treatment on the composition of mouse gut microbiome and its relationship with the cognitive abilities of mice

**DOI:** 10.1371/journal.pone.0241784

**Published:** 2020-11-18

**Authors:** Artem P. Gureev, Mikhail Yu. Syromyatnikov, Daria A. Ignatyeva, Valeria V. Valuyskikh, Sergey A. Solodskikh, Anna V. Panevina, Maria V. Gryaznova, Anastasia V. Kokina, Vasily N. Popov

**Affiliations:** 1 Department of Genetics, Cytology and Bioengineering, Voronezh State University, Voronezh, Russia; 2 Laboratory of Metagenomics and Food Biotechnology, Voronezh State University of Engineering Technologies, Voronezh, Russia; 3 Laboratory of Innovative Recombinant Proteomics, All-Russian Veterinary Research Institute of Pathology, Pharmacology and Therapy, Voronezh, Russia; McMaster University, CANADA

## Abstract

In recent years, methylene blue (MB) has attracted considerable interest as a potential drug for the treatment of methemoglobinemia and neurodegenerative diseases. MB is active against microorganisms from various taxonomic groups. However, no studies have yet been conducted on the effect of MB on the intestinal microbiome of model animals. The aim of this work was to study the effect of different concentrations of MB on the mouse gut microbiome and its relationship with the cognitive abilities of mice. We showed that a low MB concentration (15 mg/kg/day) did not cause significant changes in the microbiome composition. The *Bacteroidetes/Firmicutes* ratio decreased relative to the control on the 2^nd^ and 3^rd^ weeks. A slight decrease in the levels *Actinobacteria* was detected on the 3^rd^ week of the experiment. Changes in the content of *Delta*, *Gamma*, and *Epsilonproteobacteria* have been also observed. We did not find significant alterations in the composition of intestinal microbiome, which could be an indication of the development of dysbiosis or other gut dysfunction. At the same time, a high concentration of MB (50 mg/kg/day) led to pronounced changes, primarily an increase in the levels of *Delta*, *Gamma* and *Epsilonproteobacteria*. Over 4 weeks of therapy, the treatment with high MB concentration has led to an increase in the median content of *Proteobacteria* to 7.49% vs. 1.61% in the control group. Finally, we found that MB at a concentration of 15 mg/kg/day improved the cognitive abilities of mice, while negative correlation between the content of *Deferribacteres* and cognitive parameters was revealed. Our data expand the understanding of the relationship between MB, cognitive abilities, and gut microbiome in respect to the antibacterial properties of MB.

## Introduction

Methylene blue (MB) is an organic thiazine dye that has recently gained attention due to the newly discovered biological properties. MB is used in clinical practice to treat most forms of methemoglobinemia [1, 2]. In recent years, there has been an increasing interest in MB as a potential drug for neurodegenerative diseases such as Alzheimer's disease [3–5].

MB is active against microorganisms from various taxonomic groups and exhibits the antimalarial effect [6], as it effectively inhibits the growth of *Plasmodium falciparum*. MB was also found to exhibit *ex vivo* activity against drug-resistant isolates of *P*. *falciparum* and *Plasmodium vivax* [7]. One of the mechanisms of the antimalarial effect of MB is inhibition of glutathione reductase activity [8].

The antibacterial effect of MB in the photodynamic therapy has been well studied. The photodynamic therapy converts oxygen molecules into reactive oxygen species that act on target cells [9]. For example, MB-based photodynamic therapy reduces the amount of *Pseudomonas aeruginosa* [10]. MB-mediated photodynamic therapy effectively controls the viability of bacteria that cause dentin caries [11]. The bactericidal action of MB photodynamic therapy was shown in the destruction of both gram-negative (*Porphyromonas gingivalis* and *Aggregatibacter actinomycetemcomitans*) and gram-positive (*Streptococcus mutans*) bacteria [12]. A solution of 0.05% MB and 7% sodium citrate inhibited the growth of microorganisms such as *Escherichia coli*, *P*. *aeruginosa*, *Enterococcus faecalis*, *Staphylococcus aureus*, *Staphylococcus epidermidis*, *Candida albicans*, *Aspergillus niger*, and *Vibrio vulnificus* [13]. It has been shown that MB can be used in the treatment of periodontitis [14]. The antimicrobial effect of MB on *E*. *coli* cells has been demonstrated in zeolite [15]. A combination of citrate, MB, and parabens has a strong bactericidal effect on *S*. *aureus* biofilms [16]. Silicone with covalently bound MB exhibits a strong bactericidal activity against *E*. *coli* and *S*. *aureus* [17].

Despite the fact that MB is active against microorganisms from various taxonomic groups and already used in clinical practice, no studies have been conducted on the effect of MB on the intestinal microbiome of model animals, such as rats and mice. However, such studies are necessary due to the fact that MB can potentially affect not only harmful microorganisms, but also the useful ones that are present in the intestines of animals. It is known that changes in the composition of intestinal microbiome can lead to serious disorders including cognitive dysfunctions [18]. On one hand, MB is known to improve the cognitive abilities; on the other hand, MB has the antibacterial effect, which can lead to memory impairment due to the development of dysbiosis. Therefore, the aim of this work was to study the effect of different concentrations of MB (15 and 50 mg/kg/day) on the mouse gut microbiome and cognitive abilities of mice.

## Materials and methods

### Animals and experimental design

All experiments with animals were performed in accordance with the guidelines of the Voronezh State University Ethical Committee on Biomedical Research (Animal Care and Use Section, protocol N42-01a dated March 16, 2020). Three month-old С57Bl/6 mice were obtained from the Stolbovaya Nursery (Moscow region, Russia). The animals were kept at the 12 h light/12 h dark cycle at a temperature of 25°C - 26°C. Mice received water and a standard laboratory diet (Ssniff Spezialdiäten GmbH, Germany) *ad libitum*.

The following animal groups were used for the experiment: Control–mice that received pure water for 4 weeks (*n* = 13); MB 15 –mice that received 15 mg/kg/day MB for 4 weeks (*n* = 9); MB 50 –mice that received 50 mg/kg/day MB for 4 weeks (*n* = 8). Feces collection was performed every week during the treatment. The T-maze test was performed in the last five days of the experiment. The mice were not sacrificed after the experiment.

### T-maze test

The T-maze test for assessment of the cognitive abilities of mice was performed according to Deacon and Rawlins (2006) [18]. The height of the maze was 20 cm; the width of each arm was 10 cm; and the length of each arm was 30 cm. The mice were placed at the base of the “T” and had to choose one of the arms. In the following trail, the rodent tends to choose the opposite arm compared with the past trial. This behavioral indicator is called “spontaneous alternation”.

The experiment included two stages–habitation and trial. During the 1^st^ day of habitation, the mice moved freely throughout the maze. During the 2^nd^ day of habitation, the mice had to find food at the end of each arm. The animals were placed in a maze in groups containing mice from the same cage. Each habitation attempt lasted 3 minutes with the 10-minute intervals between the attempts. In total, each group was given 4 attempts per day.

The trials lasted 3 days. The first attempt for each mouse was training. During the training attempt, the reward was placed in each arm, but one of them remained closed. The animal was supposed to enter into the open arm and completely eat the reward. The second attempt was performed immediately, without a five-minute interval. The experimenter opened the previously closed arm, and if the animal entered the arm where it had not been before, it receives the reward. In the next attempt, the arm containing the reward was changed. If the mouse did not enter the correct arm, then in the next attempt, the reward remained in the same arm. A total of 10 attempts per day were conducted with a five-minute interval. The score corresponded to the number of correct attempts (minimum, 0; maximum, 10). The same protocol was used during all three days of trials.

### Analysis of gut microbiome composition

Feces were collected once before the start of the treatment (0 week) and at the end of each week (1^st^ to 4^th^ week). DNA was extracted from the feces with a Proba-GS kit (DNA Technology, Russia). Bacteria in the mouse feces were analyzed according to Yang et al. [19]. qPCR was performed using a Bio-Rad CFX96 Instrument (Bio-Rad, USA) and a qPCRmix-HS SYBR+LowROX kit (Evrogen, Russia). The content of bacteria of a particular phylum was determined using the following formula:

$$X=\left(E_{Univ}{}^{CqUniv}\right)/\left(E_{Spec}{}^{CqSpec}\right)\times 100\%,$$

Where, X is the percentage of bacteria of this phylum; E_Univ_ is PCR efficiency with the universal primers; E_Spec_ is PCR efficiency with the phylum-specific primers; CqUniv is the number of quantitation cycle with the universal primers; CqSpec is the number of quantitation cycle with the phylum-specific primers.

### High-throughput sequencing

To perform high-throughput sequencing, bacterial DNA isolated from feces was amplified with the universal direct 785F forward primer (5’-GGATTAGATACCCTGGTA) and reverse 1100R primer (5’-GGGTTGCGCTCGTTG) [20]. PCR was performed using a 5X ScreenMix-HS Master Mix (Evrogen, Russia) in the following regime: 94°С for 4 min followed by 37 cycles of 94°С for 30 s, 53°С for 30 s, and 72°С for 30 s with the final elongation at 72°С for 5 min. PCR products were purified with AMPure XP magnetic beads (Beckman Coulter, USA) and used for construction of sequencing libraries using Ion AmpliSeq Library Kit 2.0 (Thermo Fisher Scientific, USA) as recommended by the manufacturer. Barcoding was done using the Ion Xpress barcode adapters (Thermo Fisher Scientific, USA). Library DNA concentration was determined by qPCR using Library Quantification Kit Ion Torrent Platforms (Kapa Biosystems, USA).

Sequencing was performed with the IonTorrent PGM platform using Ion PGM Hi-Q View Sequencing Kit, Ion OneTouch 2 System, and Ion PGM Hi-Q View OT2 Kit (Thermo Fisher Scientific, USA).

The results of sequencing were obtained as binary alignment map (BAM) files that were converted into FASTQ format using the SAMtool v.1.2 software. Demultiplexing and primer stripping were done with the fastq-multx application of the ea-utils v.1.3. program package. The reads were then filtered according to the reading quality based on the number of expected errors [21, 22].

Unique sequences were identified using the DADA2 package version 1.8.0. We used negative homopolymer gap penalty value (parameter HOMOPOLYMER_GAP_PENALTY = -1), which causes homopolymer gaps to be treated as homopolymer sequences, and increased net cumulative number of insertions of one sequence relative to the other (parameter BAND_SIZE = 32).

Next, we constructed an amplicon sequence variant (ASV) table and filtered out chimeric sequences. The taxonomy (with the genus-level resolution) was assigned to the sequence variants using the DADA2 implementation of the naive Bayesian classifier method [23]. The species level taxonomy was assigned using exact matching (100% identity) with the amplicon sequence variants. Identification of bacterial genus and species was performed using the SILVA database (https://www.arb-silva.de) version 132 as a reference. We used the R version 3.4.4 for all operations related to the NGS data analysis and taxonomy assignment.

### Statistical analysis

Statistica 10 software (StatSoft, USA) was used for data analysis. The normality of the gut microbiome composition and the T-maze test score were assessed using the Shapiro-Wilk test with a significance threshold of *p* ≤ 0.05. The Mann-Whitney *U*-test was used to evaluate the differences between the experimental groups, because the Shapiro-Wilk test showed a non-normal distribution. The data were represented as the median (Q1, Q3). The Spearman's correlation was used for evaluating the association between the gut microbiome composition and the T-maze test scores.

## Results

### Gut microbiome composition

**Fig 1:**
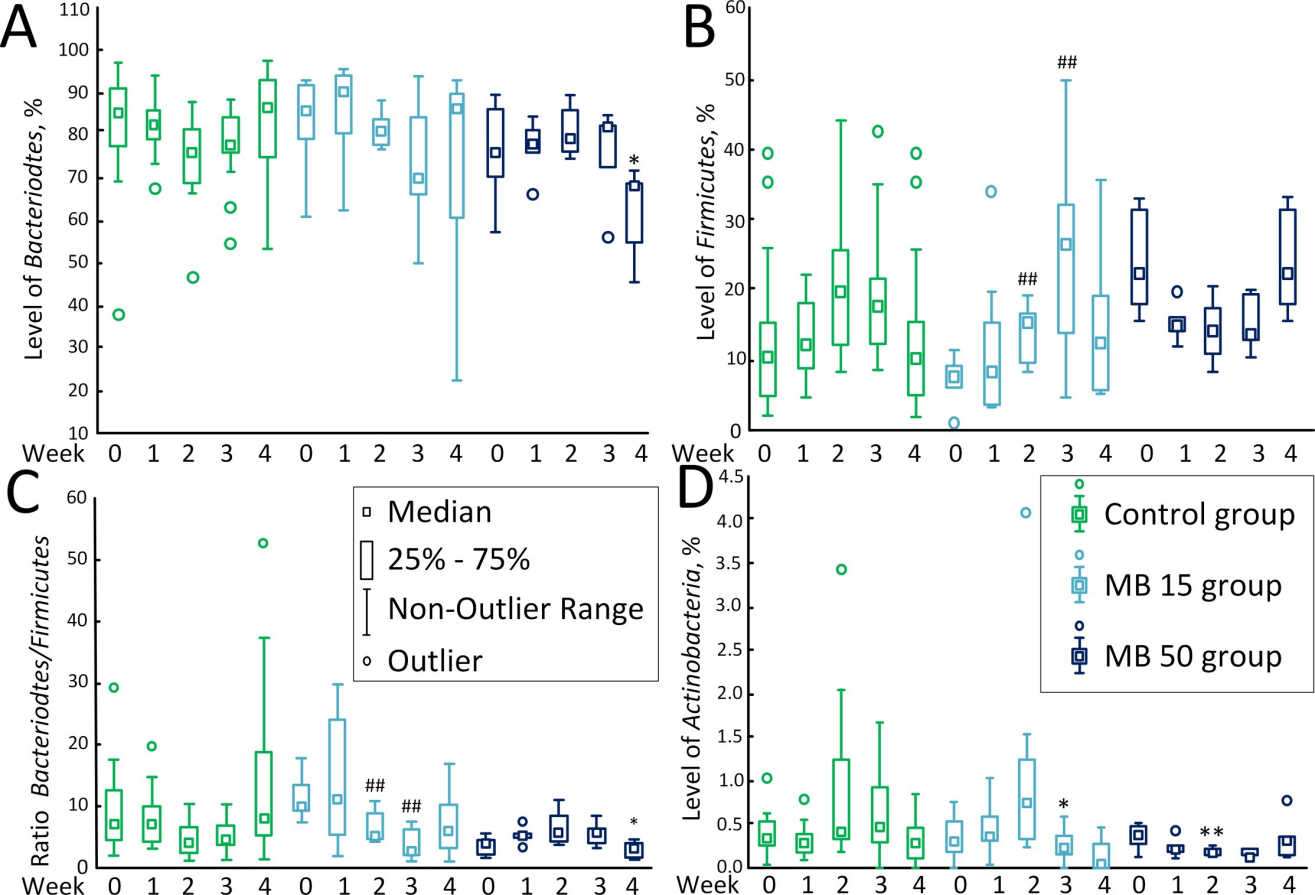
The content of predominant bacteria in the gut microbiome. (A) *Bacteroidetes*, %. (B) *Firmicutes*, %. (C) *Bacteroidetes/ Firmicutes* ratio. (D) *Actinobacteria*, %. * *p* <0.05, compared to the control on the corresponded day. ## *p* < 0.01, compared with week 0 in the corresponded experimental group.

The content of *Bacteriodetes* remained almost unaltered in the course of MB therapy. The only change was observed on the 4^th^ week of therapy in the MB 50 group, where the content of *Bacteriodetes* was 68.29% (54.97%; 68.86%) vs.86.78% (74.96%; 93.13%) in the control (*p* < 0.05) (Fig 1А).

The content of *Firmicutes* changed only in the MB 15 group. On week 0, the content of *Firmicutes* was 7.71% (5.92%; 9.08%), but by the 2^nd^ week, it grew to 15.55% (10.24%; 16.53%) (*p* <0.01), and to 26.33% (13.65%; 32.16%) on the 3^rd^ week. However, no statistically significant differences with the control group were revealed. In the MB 50 group on the 4^th^ week of therapy, the content of *Firmicutes* [22.48% (18.09%; 31.27%)] was more than two times higher than in the control group [10.46% (4.90%; 15.15%)], but the differences were statistically insignificant (*p* = 0.061) (Fig 1B). The *Bacteriodetes/Firmicutes* ratio in the MB 15 group decreased on the 2^nd^ [5.22 (4.77; 8.67); *p* < 0.01] and 3^rd^ [2.66 (2.06; 6.17); *p* < 0.01] weeks compared to week 0 [10.00 (9.32; 13.43)]. In the MB 50 group, the differences were observed only on the 4^th^ weeks, as the ratio decreased to 3.04 (1.76; 4.00) in comparison with the control [3.99 (2.25; 4.22)], (*p* < 0.05) (Fig 1C).

The level of *Actinobacteria* in the MB 15 group [0.24% (0.16%; 0.35%)] was reduced compared with the control [(0.47% (0.28%; 0.92%)] on the 3^rd^ week of therapy (*p* < 0.05) and in the MB 50 group [0.19% (0.15%; 0.21%)] vs. control [0.39% (0.30%; 1.25%)] on the 2^nd^ week of therapy (*p* < 0.01). Also, on the 2^nd^ week of therapy, a decrease in the content of *Actinobacteria* was observed in the MB 50 group compared to week 0 [0.35% (0.26%; 0.46%)], but the data were statistically insignificant (*p* = 0.094). The trend towards a decrease in the number of *Actinobacteria* in the MB 50 group compared to the control was also observed on the 3^rd^ week [0.13% (0.13%; 0.23%); *p* = 0.068] (Fig 1D).

**Fig 2:**
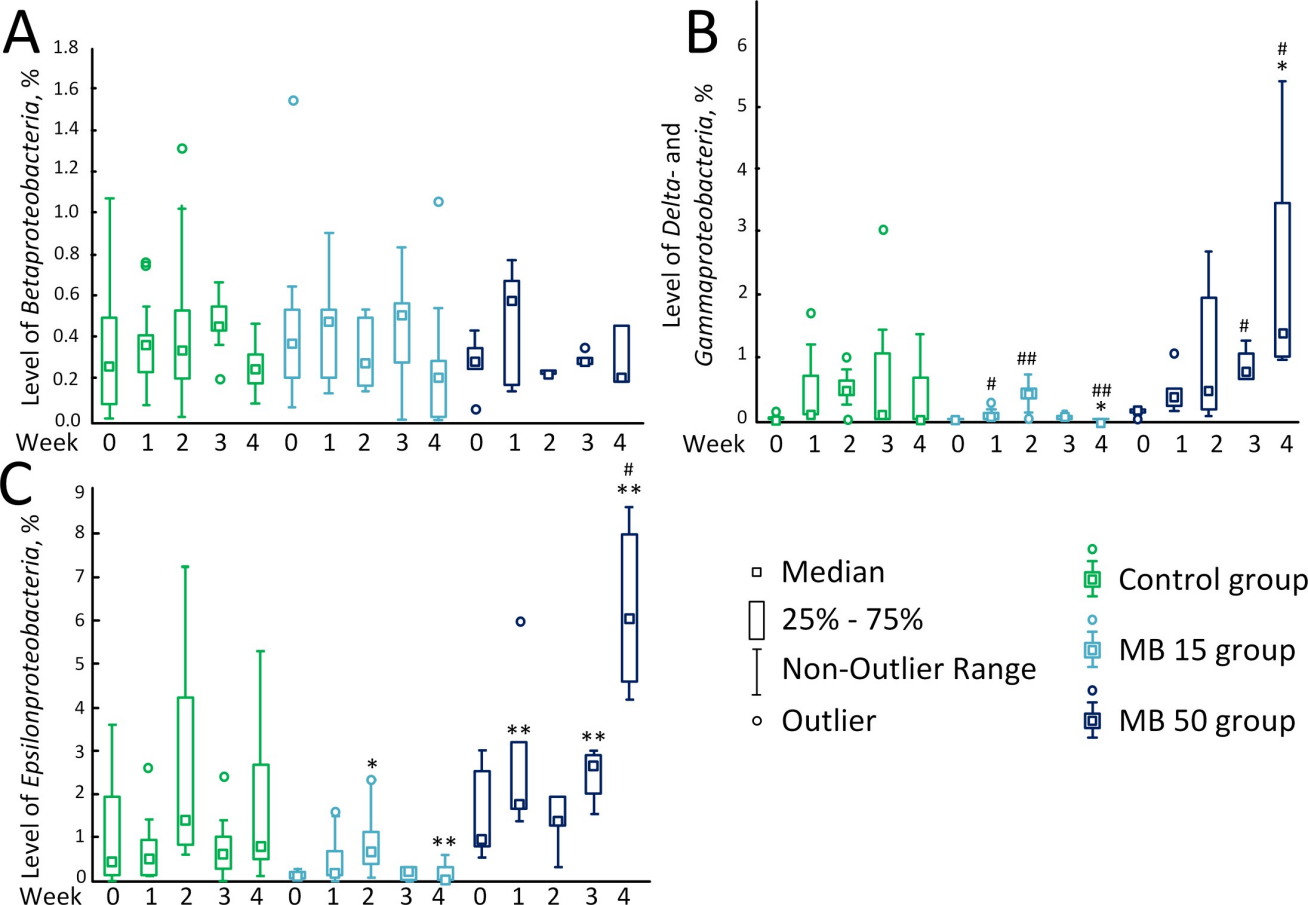
The content of predominant bacteria in the gut microbiome. (A) *Betaproteobacteria*, %. (B) *Delta*- and *Gammaproteobacteria*, %. (C) *Epsilonproteobacteria*, %. * *p* <0.05; ** *p* < 0.01, compared to the control on the corresponded day. # *p* < 0.05; ## p < 0.01, compared with week 0 in the corresponded experimental group.

The level of *Betaproteobacteria* did not change during the treatment with MB at various concentrations (Fig 2A). There were significant differences for *Delta-* and *Gammaproteobacteria*. A small number of these bacteria have been detected in the MB 15 group on week 0 [0.01% (0.01%; 0.03%)]. The number of these bacteria increased to 0.06% (0.04%; 0.12%) (*p* < 0.05) and to 0.40% (0.16%; 0.49%) (*p* < 0.01) on the 1^st^ and 2^nd^ weeks of MB treatment at a concentration of 15 mg/kg/day. However, the content of these bacteria then declined to 0.06% (0.01%; 0.08%) on the 3^rd^ week. On the 4^th^ week, *Delta-* and *Gammaproteobacteria* were not detected [0.00% (0.00%; 0.00%)]. In the MB 50 group, the level of Delta- and Gammaproteobacteria increased uniformly throughout all 4 weeks of MB therapy:week 0, 0.15% (0.13%; 0.18%); the 1^st^ week, 0.37% (0.21%; 0.51%); the 2^nd^ week, 0.46% (0.17%; 1.95%); the 3^rd^ week, 0.88% (0.68%; 1.28%); *p* < 0.05 compared with week 0; and the 4^th^ weeks, 1.40% (1.00%; 3.45%), *p* < 0.05 compared with week 0 and *p* < 0.05 compared with the control (Fig 2B).

The level of *Epsilonproteobacteria* in mice treated with MB at a concentration of 15 mg/kg/day was lower compared to the control: 0.71% (0.27%; 1.14%) vs. 1.41% (0.81%; 4.24%) in the control on the 2^nd^ week of treatment (*p* < 0.05) and 0.04% (0, 00%; 0.28%) vs. 0.82% (0.50%; 2.67%) in the control on the 4^th^ weeks (*p* < 0.01). In the MB 50 group, the content of *Epsilonproteobacteria* increased during the therapy from 0.95% (0.77%; 2.51%) on week 0 to 1.79% (1.60%; 3.18%) on the 1^st^ week (significant difference with the control, *p* < 0.01). On the 2^nd^ week of treatment, the level of *Epsilonproteobacteria* decreased to 1.39% (1.26%; 1, 92%), but increased on the 3^rd^ week to 2.68% (1.99%; 2.89%) (significant difference with the control, *p* < 0.01). On the 4^th^ week of therapy, the content of *Epsilonproteobacteria* was 6.04% (4.63%; 7.97%) (significant difference with the control, *p* < 0.01; significant difference with week 0, *p* < 0.05) (Fig 2C).

**Fig 3:**
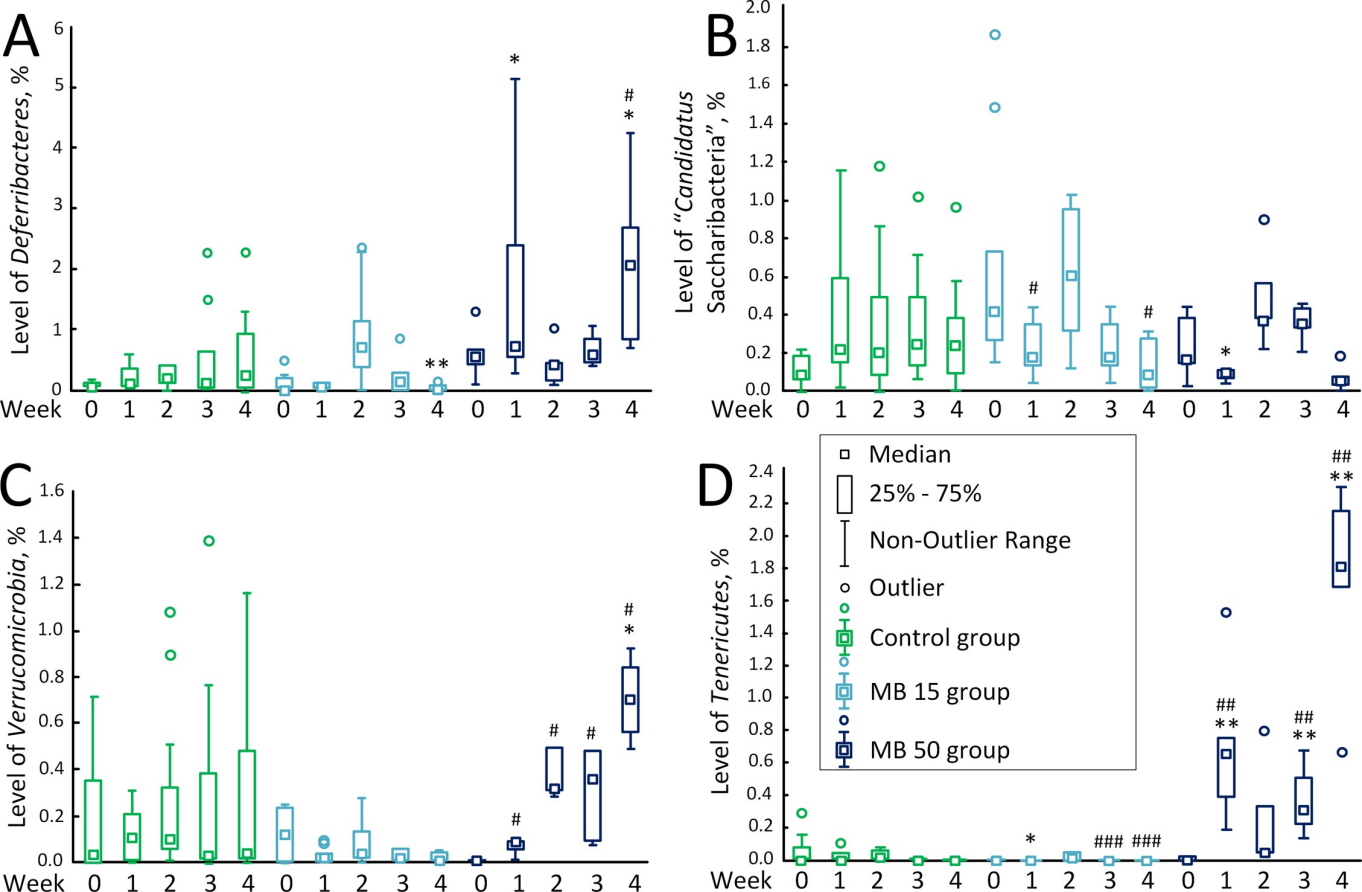
The content of predominant bacteria in the gut microbiome. (A) *Deferribacteres*, %. (B) *“Candidatus* Saccharibacteria*”*, %. (C) *Verrucomicrobia*, %. (D) *Tenericutes*, %. * *p* < 0.05; ** *p* < 0.01, compared to the control on the corresponded day. # *p* < 0.05; ## *p* < 0.01; ### *p* < 0.001, compared with week 0 in the corresponded experimental group.

The content of *Deferribacteres* in the MB 15 group (0.00% (0.00%; 0.04%) on the 4^th^ week of therapy was reduced compared to the control [0.25% (0.05%; 0.93%)] (*p* < 0.01). The number of *Deferribacteres* in the MB 50 group increased on the 2^nd^ week [0.41% (0.16%; 0.46%)] vs. control [0.11% (0.05%; 0.36%)] (*p* < 0.05) and also on the 4^th^ week of the experiment [2.07% (0.81%; 2.69%)] vs. control [0.25% (0.05%; 0.93%)] (*p* < 0.05). The content of *Deferribacteres* in the MB 50 group on the 4^th^ week of the experiment was higher than on week 0 [0.55% (0.41%; 0.66%)] (*p* < 0.05). There was also a sharp increase in the number of *Deferribacteres* [0.71% (0.25%; 1.14%)] in the MB 15 group compared to week 0 [0.01% (0.01%; 0.20%)] and control [0.21% (0.12%; 0.40%)], but the differences were statistically insignificant (*p* = 0.063 and *p* = 0.462, respectively) (Fig 3A).

The level of “*Candidatus* Saccharibacteria” fluctuated slightly in the MB 15 group; on week 0 of the experiment, the amount of “*Candidatus* Saccharibacteria” was 0.42% (0.27%; 0.73%), but already by the 1^st^ week, it decreased to 0.16% (0.13%; 0.27%) (*p* < 0.05) and then to 0.07% (0.00%; 0.23%)on the 4^th^ week (*p* < 0.05). In the MB 50 group, at the 1^st^ week of therapy, the level of “*Candidatus* Saccharibacteria” [0.09% (0.08%; 0.11%)] was lower compared to the control group [0.22% (0.15%; 0.59%)], *p* < 0.05 (Fig 3B).

In the MB 15 group, the content *Verrucomicrobia* did not change during the therapy. In the MB 50 group, the content of *Verrucomicrobia* increased uniformly throughout all 4 weeks of therapy: 0.01% (0.01%; 0.01%) on week 0; 0.09% (0.06%; 0.09%) on the 1^st^ week (*p* < 0.05 compared to the control); 0.32% (0.31%; 0.49%) on the 2^nd^ week (*p* < 0.05 compared to the control); 0.36% (0.09%; 0.48%) on the 3^rd^ week (*p* < 0.05 compared to the control); and 0.76% (0.65% 0.79%) on the 4^th^ week (*p* < 0.05 compared with week 0 and control) (Fig 3C).

The level of *Tenericutes* bacteria in the mice receiving MB at a concentration of 15 mg/kg/day was lower [0.01% (0.00%; 0.04%)] compared to the control [0.02% (0.01%; 0.06%)] on the 2^nd^ weeks of treatment (*p* < 0.05). In the course of therapy, the number of *Tenericutes* decreases by the 3^rd^ and 4^th^ weeks [0.00% (0.00%; 0.00%), *p* < 0.001]. In the MB 50 group, the content of *Tenericutes* increased from 0.01% (0.00%; 0.02%) on week to 0.66% (0.39%; 0.75%) on the 1^st^week (significant differences with week 0 and control, *p* < 0.01), decreased to 0.05% (0.05%; 0.33%) on the 2^nd^ week of treatment, and then increased again to 0.33% (0.29%; 0.61%)on the 3^rd^ week (significant differences with week 0 and control, *p* <0.01). On the 4^th^ week of therapy, the content of *Tenericutes* increased to 1.82% (1.69%; 2.16%) (significant differences with week 0 and control, *p* < 0.01) (Fig 3D).

### Species composition of bacteria in the gut microbiome

In the MB 15 group showed great variability in the number of *Bacteroidetes* within families and even genus. Among the *Alistipes* genus we showed, that MB caused increase in *A*. *obesi* (p <0.05) and *A*. *ihumii* (p <0.05), but decrease in *A*. *putredinis* (p <0.01) and *A*. *senegalensis* (p <0.01). Among the *Firmicutes* phylum in the MB 15 group were decreased level of *Lactobacillus murinus* (p <0.01), *L*. *coleohominis* (p <0.01), *Dubosiella newyorkensis* (p <0.05) and *Intestinimonas massiliensis* (p <0.01) (Table 1).

**Table 1:** MB concentration and species composition of bacteria in the gut microbiome.

Phylum	Family	Genus	Species	Control Median (Q1, Q3)	MB 15 Median (Q1, Q3)	MB 50 Median (Q1, Q3)
*Bacteroidetes*	*Muribaculaceae*	*Muribaculum*	*intestinale*	0,0492 (0,0447, 0,1107)	0,3198 (0,0447, 0,3198)	0,0860 (0,0564, 0,0919)
*Bacteroidetes*	*Bacteroidaceae*	*Bacteroides*	*acidifaciens*	0,0549 (0,0163, 0,1194)	0,0000 (0,0000, 0,0163)	0,0459 (0,0284, 0,0539)
*Bacteroidetes*	*Bacteroidaceae*	*Bacteroides*	*coprocola*	0,0000 (0,0000, 0,0000)	0,0000 (0,0000, 0,0000)	0,0000 (0,0000, 0,0000)
*Bacteroidetes*	*Bacteroidaceae*	*Bacteroides*	*caecimuris*	0,0030 (0,0016, 0,0054)	0,0021 (0,0004, 0,0026)	0,0090 (0,0077, 0,0115)
*Bacteroidetes*	*Bacteroidaceae*	*Bacteroides*	*uniformis*	0,0008 (0,0005, 0,0015)	0,0000 (0,0000, 0,0000)	0,0089 (0,0064, 0,0132)
*Bacteroidetes*	*Bacteroidaceae*	*Bacteroides*	*massiliensis*	0,0005 (0,0003, 0,0011)	0,0000 (0,0000, 0,0000)	0,0011 (0,0005, 0,0015)
*Bacteroidetes*	*Tannerellaceae*	*Parabacteroides*	*goldsteinii*	0,0008 (0,0003, 0,0027)	0,0000 (0,0000, 0,0000)	0,0036 (0,0025, 0,0043)
*Bacteroidetes*	*Tannerellaceae*	*Parabacteroides*	*johnsonii*	0,0001 (0,0000, 0,0011)	0,0000 (0,0000, 0,0000)	0,0016 (0,0013, 0,0022)
*Bacteroidetes*	*Rikenellaceae*	*Alistipes*	*obesi*	0,0000 (0,0000, 0,0000)	0,0042 (0,0032, 0,0105)	0,0000 (0,0000, 0,0000)
*Bacteroidetes*	*Rikenellaceae*	*Alistipes*	*ihumii*	0,0000 (0,0000, 0,0000)	0,0026 (0,0021, 0,0051)	0,0000 (0,0000, 0,0000)
*Bacteroidetes*	*Rikenellaceae*	*Alistipes*	*putredinis*	0,0071 (0,0051, 0,0205)	0,0000 (0,0000, 0,0000)	0,0204 (0,0152, 0,0205)
*Bacteroidetes*	*Rikenellaceae*	*Alistipes*	*senegalensis*	0,0016 (0,0015, 0,0090)	0,0000 (0,0000, 0,0000)	0,0100 (0,0089, 0,0139)
*Bacteroidetes*	*Rikenellaceae*	*Rikenella*	*microfusus*	0,0008 (0,0001, 0,0012)	0,0000 (0,0000, 0,0000)	0,0005 (0,0001, 0,0029)
*Firmicutes*	*Lachnospiraceae*	*Lachnospiraceae_*NK4A136_group	*bacterium*	0,1908 (0,0997, 0,5423)	0,1143 (0,0968, 0,3198)	0,1320 (0,0969, 0,1853)
*Firmicutes*	*Lachnospiraceae*	*Lachnospiraceae_*UCG-006	*bacterium*	0,0023 (0,0020, 0,0048)	0,0051 (0,0000, 0,0063)	0,0068 (0,0051, 0,0310)
*Firmicutes*	*Lactobacillaceae*	*Lactobacillus*	*murinus*	0,2348 (0,1450, 0,4929)	0,0000 (0,0000, 0,0000)	0,1287 (0,1015, 0,1762)
*Firmicutes*	*Lactobacillaceae*	*Lactobacillus*	*coleohominis*	0,0167 (0,0073, 0,0302)	0,0000 (0,0000, 0,0000)	0,0333 (0,0266, 0,0437)
*Firmicutes*	*Streptococcaceae*	*Streptococcus*	*danieliae*	0,0000 (0,0000, 0,0000)	0,0064 (0,0000, 0,0254)	0,0000 (0,0000, 0,0000)
*Firmicutes*	*Streptococcaceae*	*Streptococcus*	*ferus*	0,0002 (0,0000, 0,0015)	0,0000 (0,0000, 0,0000)	0,0007 (0,0004, 0,0012)
*Firmicutes*	*Streptococcaceae*	*Streptococcus*	*acidominimus*	0,0000 (0,0000, 0,0005)	0,0000 (0,0000, 0,0000)	0,0003 (0,0000, 0,0013)
*Firmicutes*	*Erysipelotrichaceae*	*Ileibacterium*	*valens*	0,0103 (0,0055, 0,0208)	0,0026 (0,0000, 0,0051)	0,0527 (0,0281, 0,0679)
*Firmicutes*	*Erysipelotrichaceae*	*Dubosiella*	*newyorkensis*	0,0060 (0,0028, 0,0093)	0,0000 (0,0000, 0,0000)	0,0268 (0,0212, 0,0348)
*Firmicutes*	*Lachnospiraceae*	*Acetatifactor*	*muris*	0,0000 (0,0000, 0,0000)	0,0000 (0,0000, 0,0000)	0,0000 (0,0000, 0,0000)
*Firmicutes*	*Erysipelotrichaceae*	*Erysipelatoclostridium*	*ramosum*	0,0000 (0,0000, 0,0000)	0,0063 (0,0000, 0,0064)	0,0000 (0,0000, 0,0000)
*Firmicutes*	*Ruminococcaceae*	*Intestinimonas*	*massiliensis*	0,0035 (0,0019, 0,0037)	0,0000 (0,0000, 0,0000)	0,0076 (0,0049, 0,0324)
*Firmicutes*	*Erysipelotrichaceae*	*Faecalibaculum*	*rodentium*	0,0025 (0,0014, 0,0047)	0,0000 (0,0000, 0,0051)	0,0014 (0,0006, 0,0016)
*Actinobacteria*	*Bifidobacteriaceae*	*Bifidobacterium*	*animalis*	0,0000 (0,0000, 0,0000)	0,0318 (0,0051, 0,0323)	0,0000 (0,0000, 0,0000)
*Actinobacteria*	*Bifidobacteriaceae*	*Bifidobacterium*	*pseudolongum*	0,0000 (0,0000, 0,0000)	0,0000 (0,0000, 0,0000)	0,0000 (0,0000, 0,0000)
*Actinobacteria*	*Propionibacteriaceae*	*Cutibacterium*	*acnes*	0,0000 (0,0000, 0,0000)	0,0000 (0,0000, 0,0000)	0,0000 (0,0000, 0,0000)
*Actinobacteria*	*Eggerthellaceae*	*Enterorhabdus*	*caecimuris*	0,0010 (0,0004, 0,0012)	0,0106 (0,0000, 0,0132)	0,0014 (0,0010, 0,0020)
*Actinobacteria*	*Corynebacteriaceae*	*Corynebacterium_1*	*stationis*	0,0005 (0,0000, 0,0040)	0,0000 (0,0000, 0,0000)	0,0005 (0,0001, 0,0008)
*Proteobacteria*	*Pasteurellaceae*	*Rodentibacter*	*pneumotropicus*	0,0000 (0,0000, 0,0002)	0,0000 (0,0000, 0,0000)	0,0015 (0,0006, 0,0016)
*Proteobacteria*	*Sutterellaceae*	*Parasutterella*	*excrementihominis*	0,0016 (0,0005, 0,0024)	0,0211 (0,0152, 0,0323)	0,0054 (0,0018, 0,0152)
*Proteobacteria*	*Moraxellaceae*	*Enhydrobacter*	*aerosaccus*	0,0000 (0,0000, 0,0002)	0,0000 (0,0000, 0,0000)	0,0000 (0,0000, 0,0000)
*Proteobacteria*	*Oxalobacteraceae*	*Massilia*	*aurea*	0,0000 (0,0000, 0,0003)	0,0000 (0,0000, 0,0000)	0,0000 (0,0000, 0,0000)
*Proteobacteria*	*Sutterellaceae*	*Sutterella*	*wadsworthensis*	0,0000 (0,0000, 0,0000)	0,0000 (0,0000, 0,0000)	0,0000 (0,0000, 0,0000)
*Proteobacteria*	*Neisseriaceae*	*Snodgrassella*	*alvi*	0,0000 (0,0000, 0,0000)	0,0032 (0,0000, 0,0042)	0,0000 (0,0000, 0,0000)
*Epsilonbacteraeota*	*Helicobacteraceae*	*Helicobacter*	*apodemus*	0,0693 (0,0294, 0,0974)	0,1907 (0,1613, 0,2763)	0,2622 (0,1451, 0,4093)
*Epsilonbacteraeota*	*Helicobacteraceae*	*Helicobacter*	*ganmani*	0,0042 (0,0027, 0,0055)	0,0000 (0,0000, 0,0102)	0,0084 (0,0052, 0,0134)
*Epsilonbacteraeota*	*Helicobacteraceae*	*Helicobacter*	*mastomyrinus*	0,0040 (0,0027, 0,0065)	0,0000 (0,0000, 0,0000)	0,0099 (0,0058, 0,0128)
*Deferribacteres*	*Deferribacteraceae*	*Mucispirillum*	*schaedleri*	0,0084 (0,0048, 0,0110)	0,0297 (0,0152, 0,0645)	0,0314 (0,0294, 0,0508)

In the MB 50 group was observed a 10-fold increase in the level of *Bacteroides uniformis* (p <0.01) compared with control and 5-fold increase in the level of *Ileibacterium valens* (p <0.01). We observed that level of some *Proteobacteria* was increased compared to the control in MB 50 group the 4^th^ week of therapy. It was showed for *Helicobacter apodemus* (*Epsilonbacteraeota*) (p <0.05)) and *Rodentibacter pneumotropicus* (Gammaproteobacteria) (p <0.05)). Also, we showed 4-fold increase in the level of *Mucispirillum schaedleri* (Deferribacteres) (p <0.01) (Table 1).

### T-maze test results

**Fig 4:**
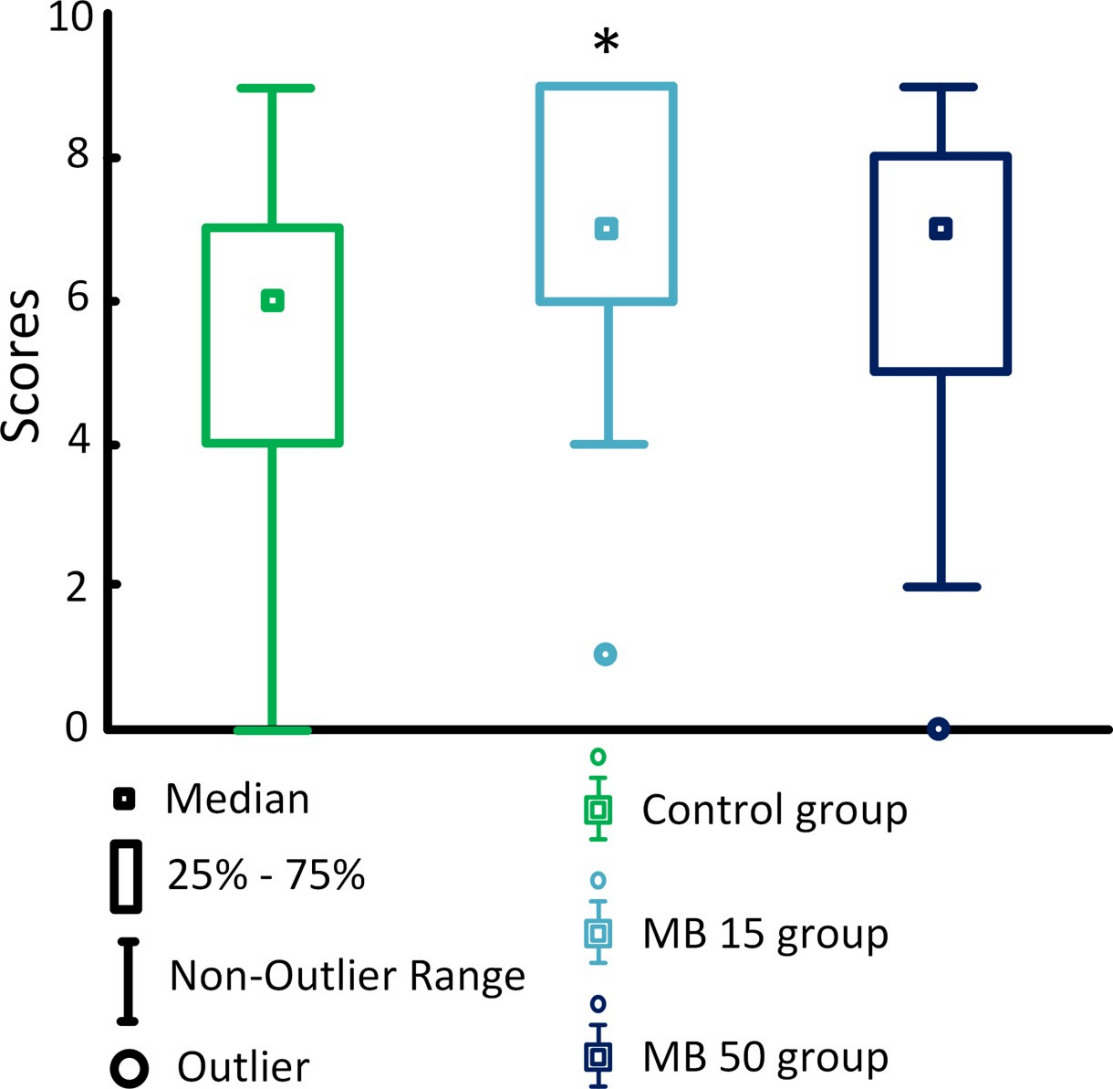
Scores in all trials of the T-maze test. * *p* < 0.05, compared with the control.

MB at a concentration of 15 mg/kg/day improved the memory of mice compared to the control [score 7 (6; 9) for the MB 15 group versus score 6 (4; 7) in the control, *p* < 0.01], while no differences were detected for the mice treated with MB at a concentration of 50 mg/kg/day [score 5 (7; 8)] (Fig 4).

On the first day of the trials, the highest results were demonstrated by the MB 50 group [score 7 (6; 8)); the score for the MB 15 group was lower (score 7 (6; 7)], while the control group demonstrated significantly lower score [score 5 (3; 7)]. However, on the second day of trails, the highest results were demonstrated by the MB 15 group [score 7 (5; 9)], while the other two groups had lower scores: score 5 (3; 8) in the MB 50 group and score 5 (4; 6). in the control group. On the third day, the highest scores were also shown by the MB 15 group [score 9 (8; 9)], the scores for the MB 50 and control groups were 7 (6; 7) and 7 (6; 9), respectively.

### Correlation analysis

We also found the correlations between most groups of the studied microorganisms. The content of *Bacteroidetes* negatively correlated with the content of *Firmicutes* (*r*_s_ = -0.611, *p* < 0.05), *Delta-* and *Gammaproteobacteria* (*r*_s_ = -0.425, *p* < 0.05), and *Epsilonproteobacteria* (*r*_s_ = -0.434, *p* < 0.05). On the contrary, the content of *Firmicutes* positively correlated with the levels of *Delta-and Gammaproteobacteria* (*r*_s_ = 0.594, p < 0.05), *Epsilonproteobacteria* (*r*_s_ = 0.570, p < 0.05), and *Actinobacteria* (*r*_s_ = 0.450, p < 0.05). The highest correlation was observed between the levels of *Delta-* and *Gammaproteobacteria* and *Epsilonproteobacteria* (*r*_s_ = 0.852, *p* < 0.05). The content of *Deferribacteres* negatively correlated with the level of *Bacteroidetes* (*r*_s_ = -0.385, *p* < 0.05). A positive correlation was found between the content of *Deferribacteres* and the levels of *Firmicutes* (*r*_s_ = 0.554, *p* < 0.05), *Actinobacteria* (*r*_s_ = 0.555, *p* < 0.05), *Delta-* and *Gammaproteobacteria* (*r*_s_ = 0.864, *p* < 0.05), and *Epsilonproteobacteria* (*r*_s_ = 0.925, *p* < 0.05). Similarly, the content of *Tenericutes* positively correlated with the level of *Bacteroidetes* (*r*_s_ = -0.385, *p* < 0.05) and negatively correlated with the levels of *Firmicutes* (*r*_s_ = 0.639, *p* < 0.05), *Delta-* and *Gammaproteobacteria* (r_s_ = 0.765, p < 0.05), *Epsilonproteobacteria* (*r*_s_ = 0.847, *p*<0.05), and *Deferribacteres* (*r*_s_ = 0.798, *p* < 0.05). The levels of *Verrucomicrobia* positively correlated with the levels of *Firmicutes* (*r*_s_ = 0.520, *p* < 0.05), *Actinobacteria* (*r*_s_ = 0.420, *p* < 0.05), *Delta-* and *Gammaproteobacteria* (*r*_s_ = 0.781, *p* < 0.05), *Epsilonproteobacteria* (*r*_s_ = 0.779, p < 0.05), *Deferribacteres* (*r*_s_ = 0.789, *p* < 0.05), and *Tenericutes* (*r*_s_ = 0.790, *p* < 0.05). The lowest correlation was found for the phylum *Betaproteobacteria* [its content correlated only with the level of *Bacteroidetes* (*r*_s_ = 0.380, *p* < 0.05)] and “*Candidatus* Saccharibacteria” [correlated only with the level of *Actinobacteria* (*r*_s_ = 0.406, *p* < 0.05)] (Table 2).

**Table 2:** Correlation between gut microbiome composition and results of T-maze test.

	B	F	A	β	γ	ε	D	S	T	V	1^st^ d s	2^nd^d s	3^rd^d s	M s
Bac		**-0,611**	-0,294	**0,380**	**-0,425**	**-0,434**	**-0,385**	0,237	**-0,553**	-0,293	0,113	-0,166	-0,157	-0,136
Firm			**0,450**	-0,043	**0,594**	**0,570**	**0,554**	0,050	**0,639**	**0,520**	-0,044	-0,082	0,008	-0,038
Act				0,225	**0,448**	**0,545**	**0,555**	**0,406**	0,356	**0,420**	-0,250	-0,222	-0,080	-0,199
Beta					0,174	0,236	0,262	-0,020	0,001	0,279	0,360	0,013	-0,143	0,077
Gamma						**0,852**	**0,864**	-0,005	**0,765**	**0,781**	0,000	-0,241	-0,119	-0,083
Epsilon							**0,925**	0,045	**0,847**	**0,779**	-0,167	-0,350	-0,231	-0,264
Defer								0,127	**0,798**	**0,789**	-0,151	**-0,442**	-0,288	-0,316
Sac									0,004	0,073	-0,190	-0,290	-0,291	-0,334
Ten										**0,790**	-0,096	-0,247	-0,321	-0,195
Ver											-0,037	-0,246	**-0,406**	-0,250
1^st^ d s												**0,514**	0,270	**0,705**
2^nd^ d s													**0,705**	**0,895**
3^rd^ d s														**0,781**
M s														

B–Bac–*Bacteroidetes*; F–Firm–*Firmicutes*; A–Act–*Actinobacteria*; β–Beta–*Betaproteobacteria*; γ—*Gamma—Delta- and Gammaproteobacteria*; ε–Epsilon–*Epsilonproteobacteria*; D–Defer–*Deferribacteres*; S–Sac—“*Candidatus* Saccharibacteria”; T–Ten–*Tenericutes*; V–Ver–*Verrucomicrobia*; 1^st^ d s–First day score; 2^nd^ d s—Second day score; 3^rd^ d s–Third day score. **Bold font**—the correlation is statistically significant, p<0.05.

Regarding to the T-maze tests, there was a strong connection between the scores obtained on different days. The average score correlated most strongly with the scores of the 2^nd^ day of the trials (*r*_s_ = 0.895, *p* < 0.05) and somewhat less with the scores of the 3^rd^ day (*r*_s_ = 0.781, *p* < 0.05) and the 1^st^ day (*r*_s_ = 0.705, *p* < 0.05) of trials (Table 2).

**Fig 5:**
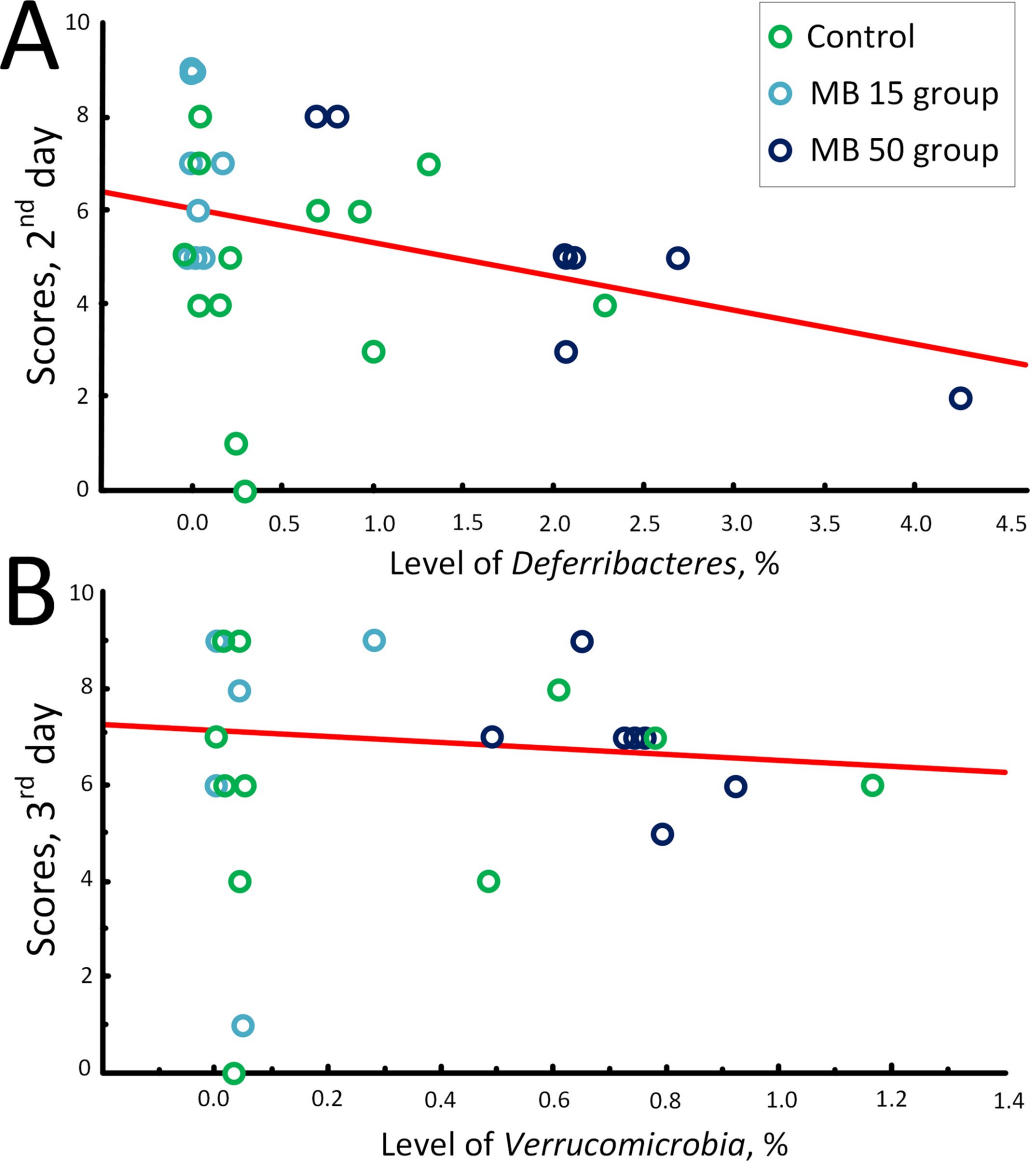
Correlation between the T-maze test scores and content of bacterial groups on the 4^th^ week of treatment. (A) Correlation between the T-maze test scores in (2^nd^ day) and content of *Deferribacteres*, %; (B) Correlation between the T-maze test scores (3^rd^ day) and content of *Verrucomicrobia*, %.

However, the correlation between the levels of bacteria in the intestinal microbiome and the results of the T-maze test was extremely low. The negative correlation was found only between the level of *Deferribacteres* and the scores obtained on the 2^nd^ day of the trials (*r*_s_ = -0.442, *p* < 0.05) (Table 1, Fig 5A) and the level of *Verrucomicrobia* and the scores obtained on the 3^rd^ day of trials (*r*_s_ = -0.406, *p* < 0.05) (Table 2, Fig 5B).

## Discussion

The question about the optimal MB concentration of for the treatment of animals is largely debatable. The studies in rats have shown that the optimal concentration for the injection is 1 to 4 mg/kg [24, 25]. Single injections of 50–100 mg/kg MB suppressed the running wheel behavior [26] but did not cause a genotoxic effect [27]. When MB was taken orally with drinking water, a positive effect was observed in the MB concentration range from 15 to 40 mg/kg/day [28–30]. There is no information on any adverse effects of MB when used at the concentrations above 40 mg/kg/day.

No systematic studies have been conducted to evaluate possible negative effect of MB on the gastrointestinal tract. In contrast, MB was found to demonstrate the therapeutic effect against ulcerative colitis induced by intrarectal administration of 2,4,6-trinitrobenzene sulfonic acid [31] and against acetic acid-induced colitis in colonic mucosa [32]. However, a single case was reported when a standard procedure of submucosal injection of 0.01% MB during colon resection induced acute localized colitis [33]. Although it has been shown earlier that chromoendoscopy with MB is a potent tool for the early detection of intraepithelial neoplasias and colon carcinomas in patients with ulcerative colitis [34–38].

We showed that low MB concentrations (15 mg/kg /day) did not cause significant changes in the gut microbiome composition. The level of *Firmicutes* has occasionally increased, but only in comparison with week 0, but not with control (Fig 1B). The *Bacteroidetes/Firmicutes* ratio decreased relative to the control on the 2^nd^ and 3^rd^ weeks (the tendency to decrease was also observed on the 4^th^ week, *p* = 0.063) (Fig 1C). This is consistent with data from Manderino et al. (2017), who showed that people with a higher cognitive performance have a reduced content of *Bacteroidetes* and increased content of *Firmicutes* [39]. A slight decrease in the level of *Actinobacteria* was detected on the 3^rd^ week of experiment. The changes in the content of *Delta*, *Gamma*, and *Epsilonproteobacteria* have been also observed (Fig 2B and 2C). However, these changes were rather of the “oscillatory nature” and did not exceed 1%. Similar oscillatory changes within 1% caused by MB at a concentration of 15 mg/kg/day were also observed for *Deferribacteres* and “*Candidatus* Saccharibacteria” (Fig 3A and 3B). However, in general, we found no significant changes in the intestinal microbiome composition, which could be a marker of the development of dysbiosis or other gut dysfunction.

At the same time, high MB concentrations (50 mg/kg/day) led to significant changes in the composition of gut microbiome, mostly, an increase in the levels of *Delta*, *Gamma* and *Epsilonproteobacteria* (Fig 2B and 2C). Within 4 weeks of therapy, the content of *Proteobacteria* increased to 7.49% (6.05%; 12.26%) vs. 1.61% (0.80%; 3.96%). in the control group. Sequencing showed that high concentration of MB caused increase in *Helicobacter apodemus* (Epsilonbacteraeota) and *Rodentibacter pneumotropicus* (Gammaproteobacteria). Not all species of the *Helicobacter* genus are pathogenic, but an increase of these bacteria suggests that there may be damage to organs or organ systems [40]. An increase in the level of bacteria of the genus *Rodentibacter* may indicate the presence of an infection [41]. Recently, Danilova et al. [42] showed that the increase in the content of *Proteobacteria* in the microbiome is a marker of the development of inflammatory bowel disease (which includes Crohn's disease and ulcerative colitis). Other indicators of the of inflammatory bowel disease development are an increase in the number of *Bacteroidetes* and decrease in the number of *Firmicutes* [42]. However, in our study, on the contrary, we found a decrease in the level of *Bacteroidetes* on the 4^th^ week of the high-dose MB treatment (Fig 1A). No effect on *Firmicutes* was detected (Fig 1B). Therefore, even if the content of *Proteobacteria* increased, we cannot unequivocally conclude that high MB concentrations cause changes in the intestinal microbiome typical for patients with inflammatory bowel disease.

The content of *Proteobacteria* increases in dysbiosis [18]. There is no consensus in medicine whether dysbiosis is a consequence or a cause of the inflammatory bowel disease [43]. It is possible that the long-term treatment with high MB concentrations can have a negative effect on the gut microbiome. Another change that can be characterized as negative was an increase in the level of *Deferribacteres* (Fig 3A). In particular, we showed 4-fold increase in the level of *Mucispirillum schaedleri* (Deferribacteres). *M*. *schaedleri* is a pathobiont, commensal, which plays a role in the development of the disease and their increase in the body means the presence of intestinal inflammation in the studied organism [44].

Usually, an increase in the content of *Deferribacteres* is associated with the development of inflammatory processes, as *Deferribacteres* have been suggested to be mucus-dwelling commensals that can cause the disease [45]. A connection between inflammation and increase in the level of *Proteobacteria* has already been shown [46]; therefore, we assume that the development of the MB-induced dysbiosis can cause inflammatory processes that can adversely affect the functioning of the whole organism, in particular, the brain and its cognitive functions [47].

A relationship between intestinal dysbiosis and development of inflammation due to the loss of mucosal surfaces has been established [48]. Chronic inflammation is considered an important factor in the cognitive decline [49]. It has been shown repeatedly that the increase in the content of *Proteobacteria* is associated with certain cognitive deficits [39, 50].

We found that the MB therapy at a concentration of 15 mg/kg/day improved the cognitive parameters in mice, what was manifested as higher scores in the spontaneous alternation test (Fig 4). Alternation reflects motivation of the mice to find food. The T-maze alternation is one of most popular tests for the evaluation of cognitive abilities of rodents [51].

Most likely, this effect was achieved due to the unique properties of MB, which can act as an alternative electron carrier [52]. It has been repeatedly shown that MB improves memory in various experimental models [53–57]. At the same time, we showed that at a concentration of 50 mg/kg/day, MB did not significantly increase the cognitive parameters of mice. Probably, one of the reasons why the neurostimulating properties of MB were suppressed in this case was the development of dysbiosis and subsequent inflammatory process. We found a negative correlation between the level of *Deferribacteres* and test scores on the 2^nd^ day of trials in the T-maze test (*r*_s_ = -0.44, *p* < 0.05) (Table 1, Fig 5A), which was consistent with the suggestion that an increase in the content of *Deferribacteres* is associated with inflammatory process [45] negatively affecting cognitive functions [49].

However, it was previously shown that the level of *Verrucomicrobia* positively correlates with the cognitive function in neurologically healthy older adults [39], which contradicted our data, as we demonstrated negative correlation with the scores on the 3^rd^ day of trials in the T-maze test (*r*_s_ = -0.41, *p* < 0.05) (Table 1, Fig 5B). Probably, the role of *Verrucomicrobia* in the microbiome relationship with cognitive properties is species-specific, and it is impossible to unambiguously extrapolate the cognitive characteristics of mice onto cognitive characteristics of humans. But the correlation analysis does not guarantee that there is a direct link between microbiome composition and behavioral parameters. This analysis was carried out in order to "outline" possible relationships that could prove or disprove subsequent research. Moreover, the T-maze test does not allow a full assessment of the cognitive abilities of mice, and more tests are required to unambiguously assess the effect of MB on cognitive functions, for example, the Morris water maze. In addition, it was interesting in the future to evaluate MB effect on the gut microbiome and cognitive ability of mice of different ages, not only three-month-old mice, which did not have chronic disease.

In summary, we found that MB treatment in a low dose (15 mg/kg/day) improved the cognitive abilities of mice. In contrast, MB treatment in a high dose (50 mg/kg/day) did not affect the cognitive abilities. We suggest that this might be due to the development of dysbiosis mediated by the increase in the content of *Proteobacteria* and *Deferribacteres*. Moreover, we revealed a negative correlation between the level of *Deferribacteres* and scores in the T-maze test. Our data expand the understanding of the relationship between MB, cognitive abilities, and gut microbiome in relation to the antibacterial properties of MB.

## Supporting information

S1 Table Gut micriobiome composition (%) for each mouse in all experimental groups. (XLSX)

S2 Table Scores for each mouse in all experimental groups in the T-maze test. (XLSX)

S3 Table Table of bacterial taxa after the high-throughput sequencing. (XLSX)

S1 File Box-plot graphs obtained using Statistica 10 software for gut microbiome composition. (DOCX)
