# Supplementary figures and images for: Effect of long-term methylene blue treatment on the composition of mouse gut microbiome and its relationship with the cognitive abilities of mice
Source: PLoS One. 2020 Nov 18;15(11):e0241784. doi: 10.1371/journal.pone.0241784 (PMC7673545; doi:10.1371/journal.pone.0241784)

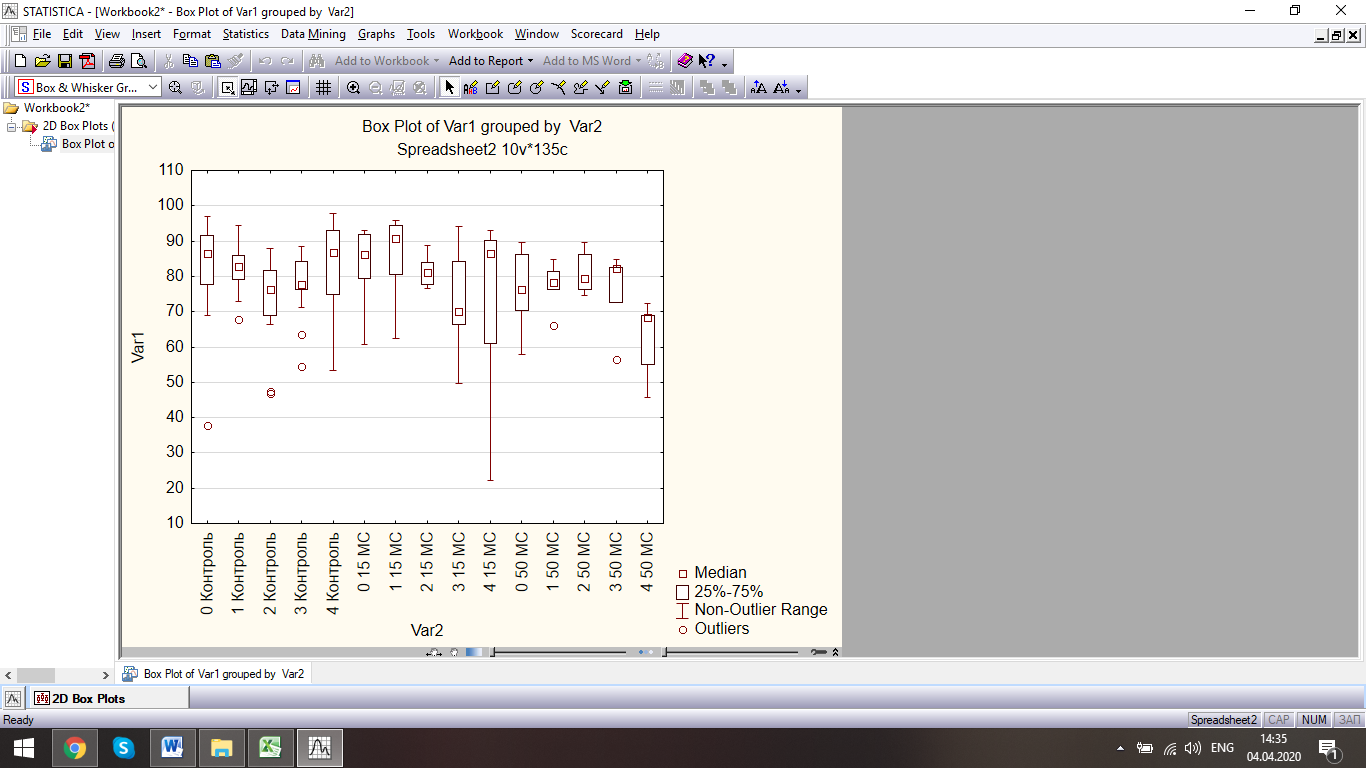


**Вас**


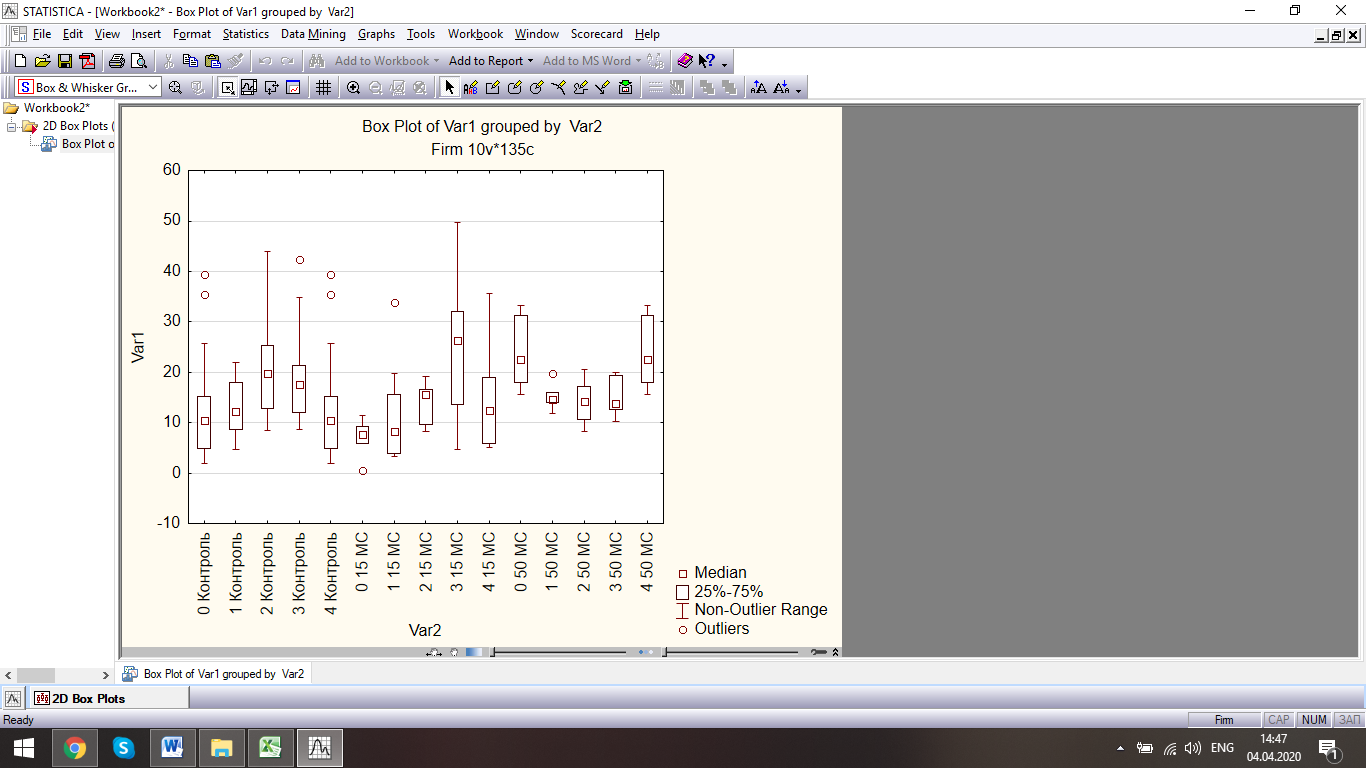


Firm


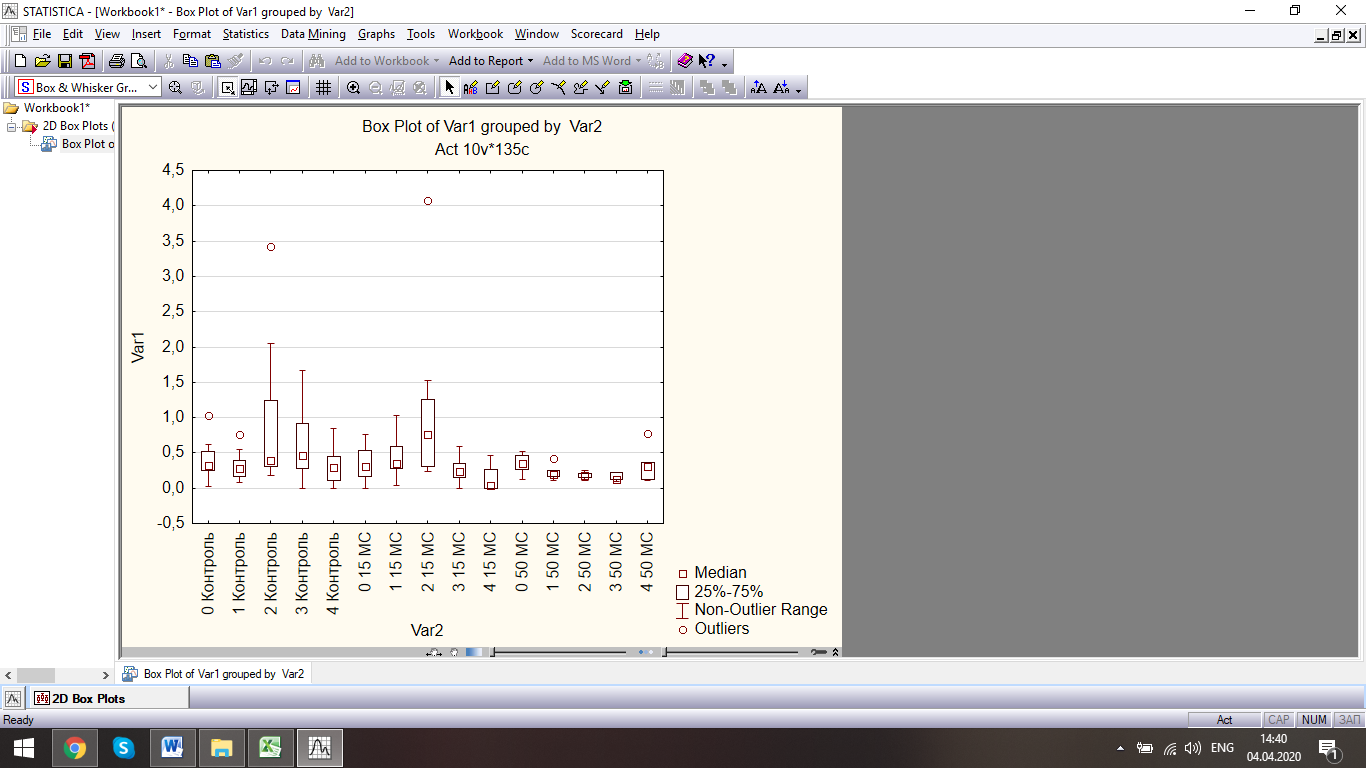


Act

Beta


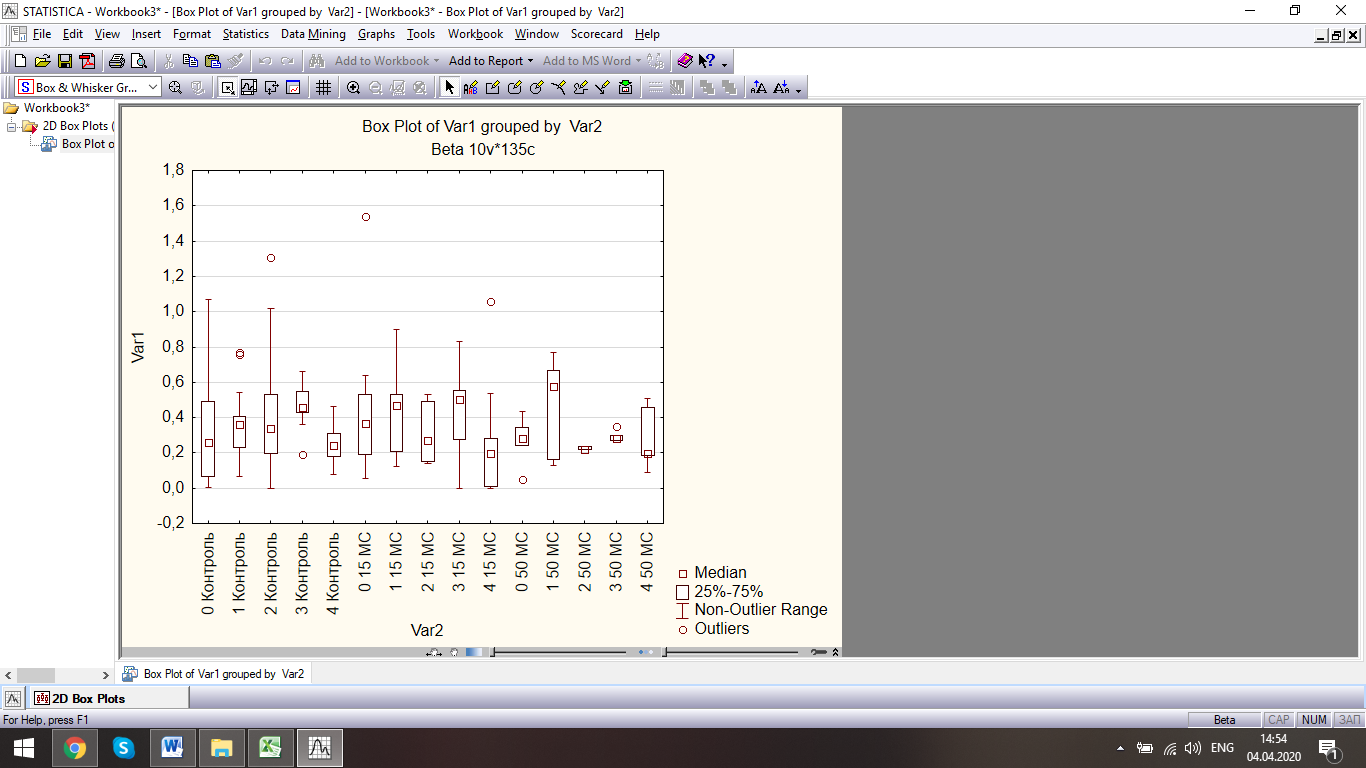


Gamma


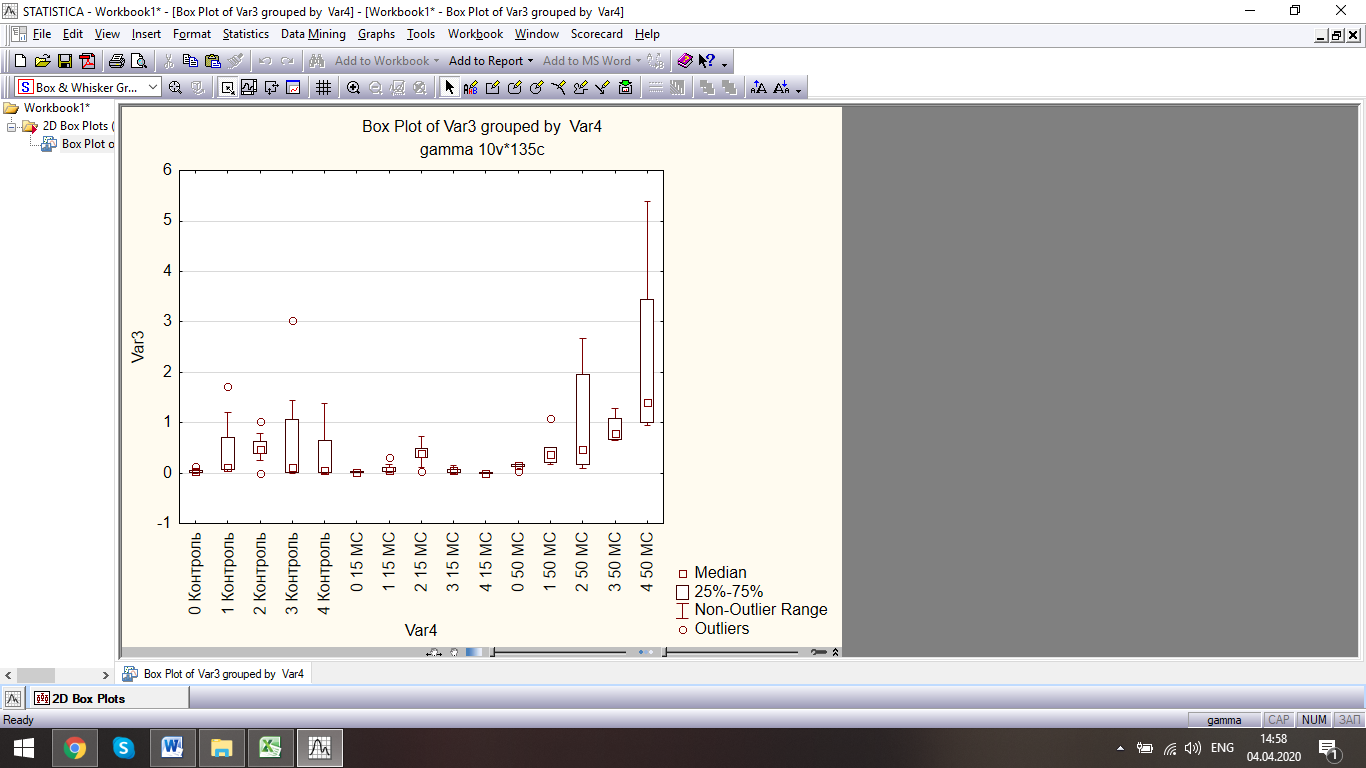


Epsilon


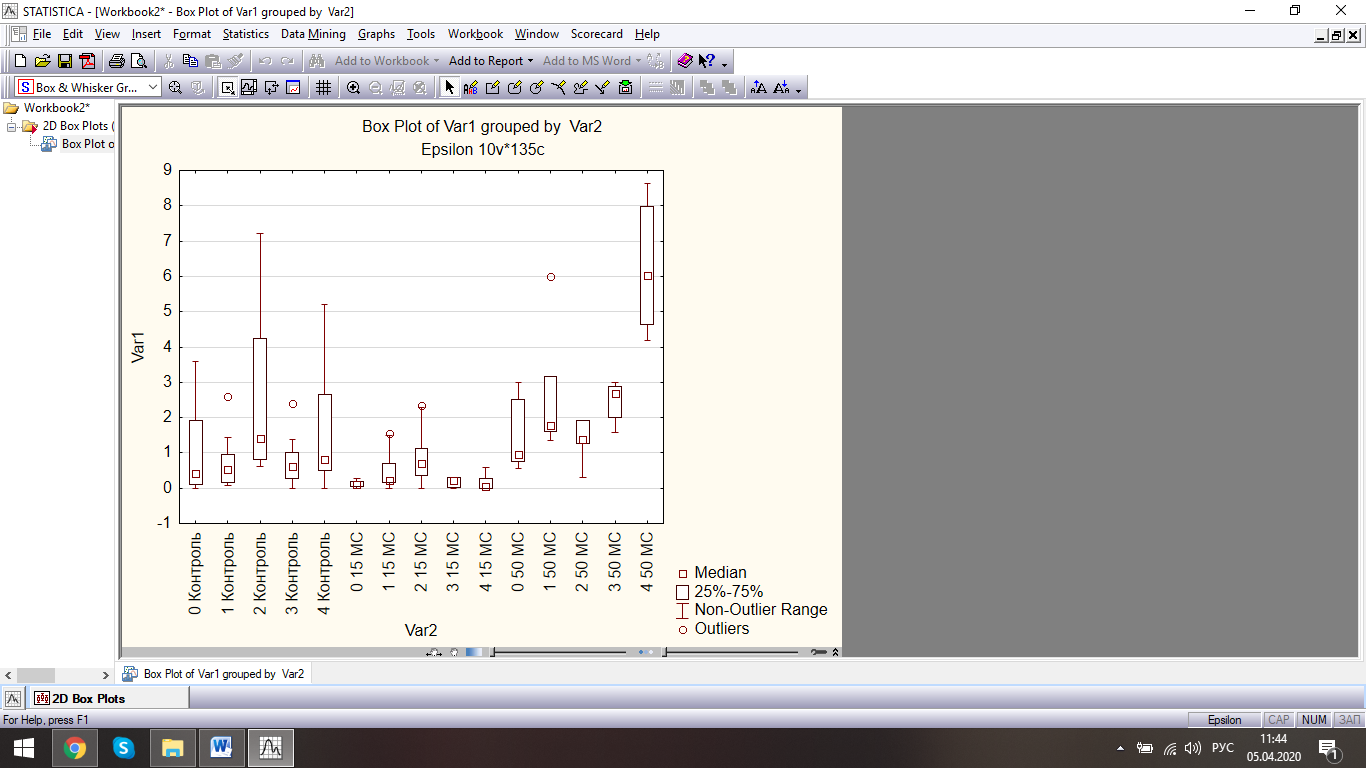


**Defer**


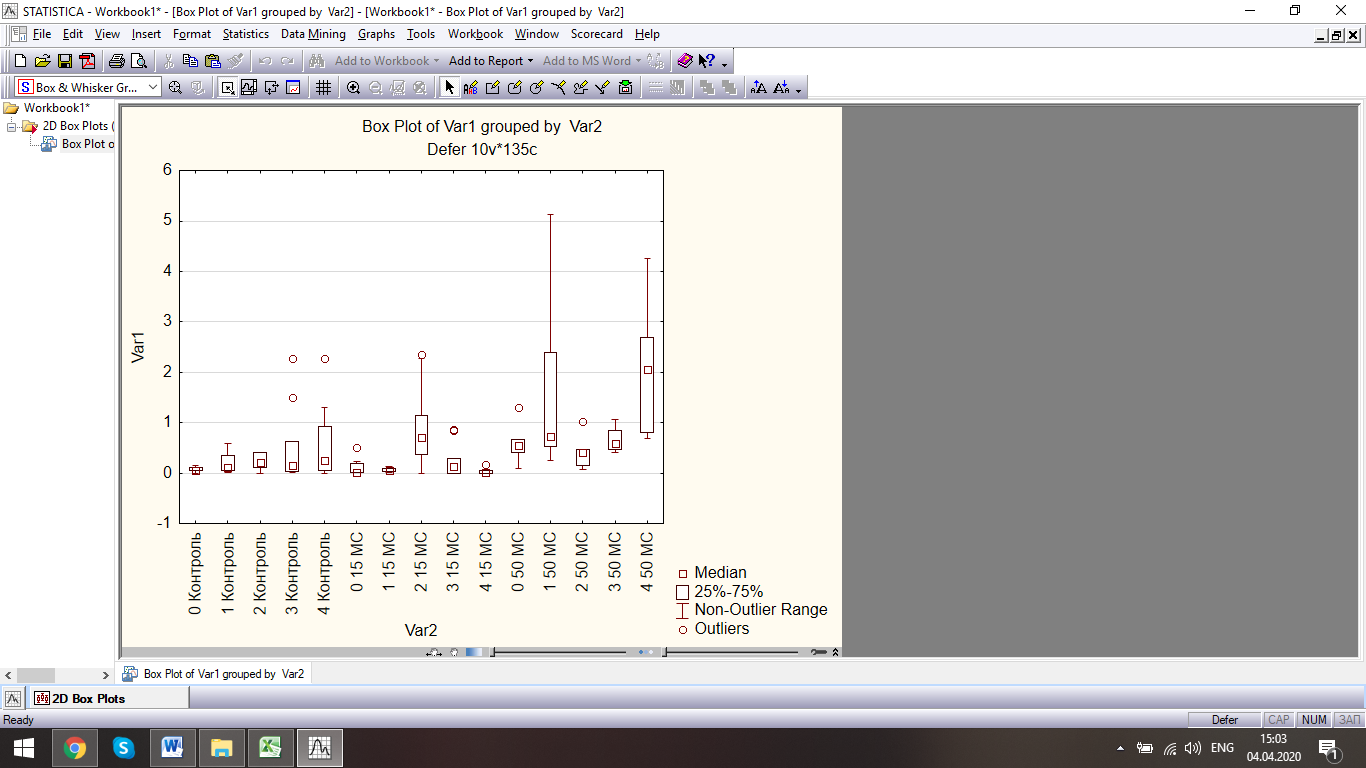


Sac


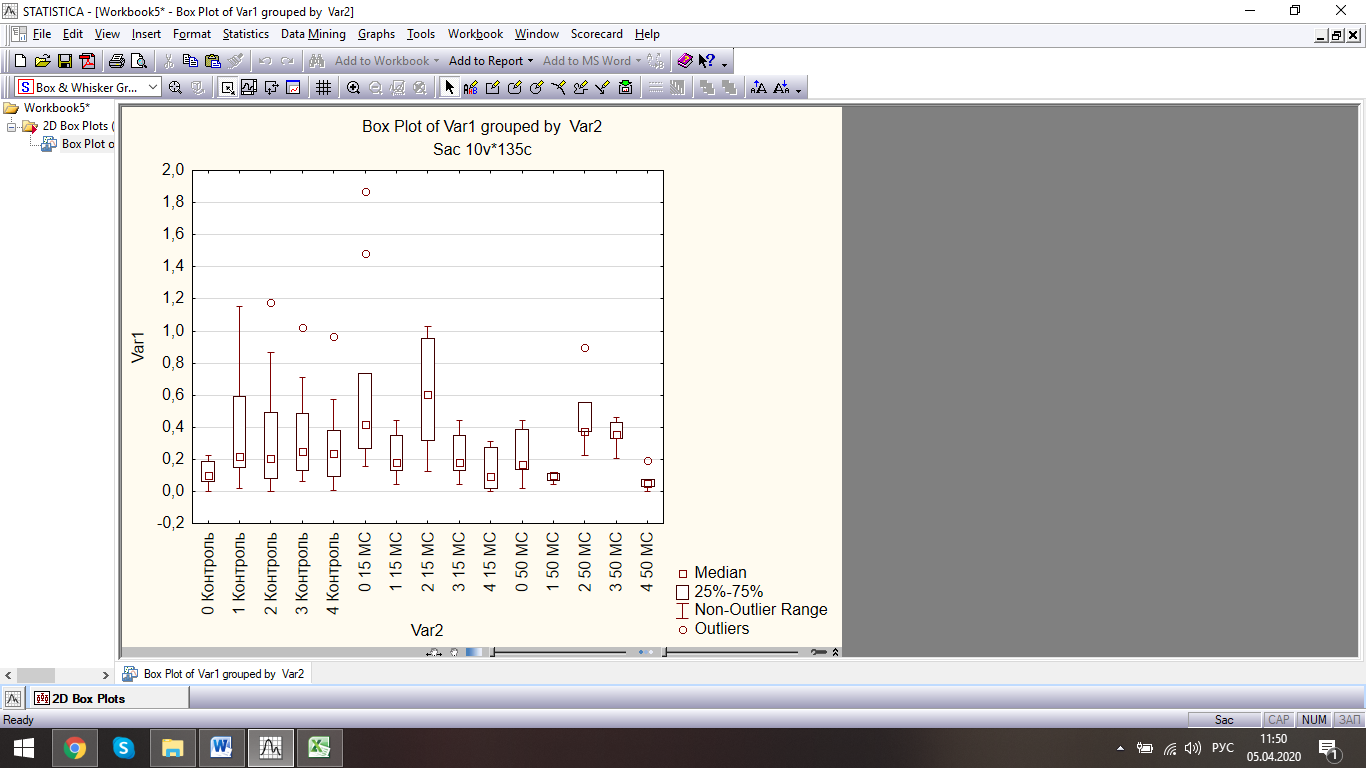


Ten


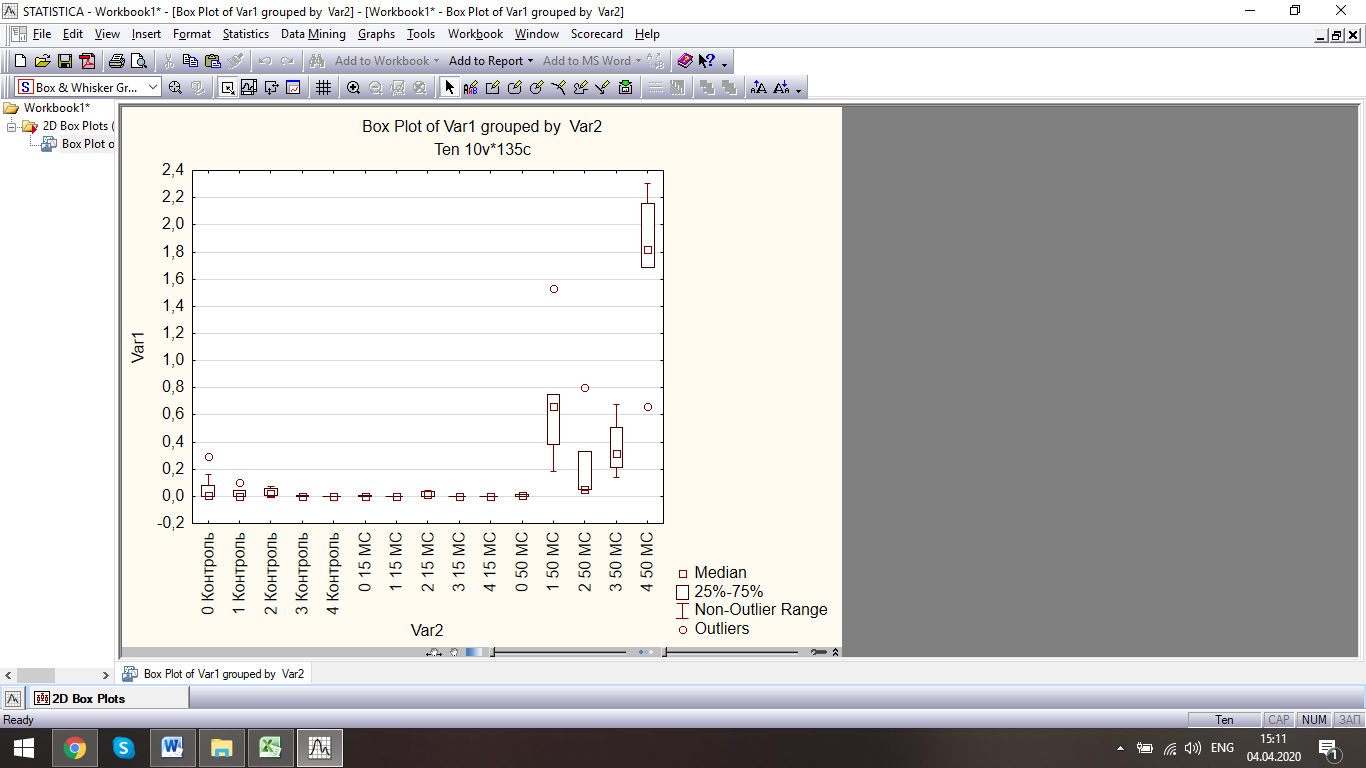


Ver


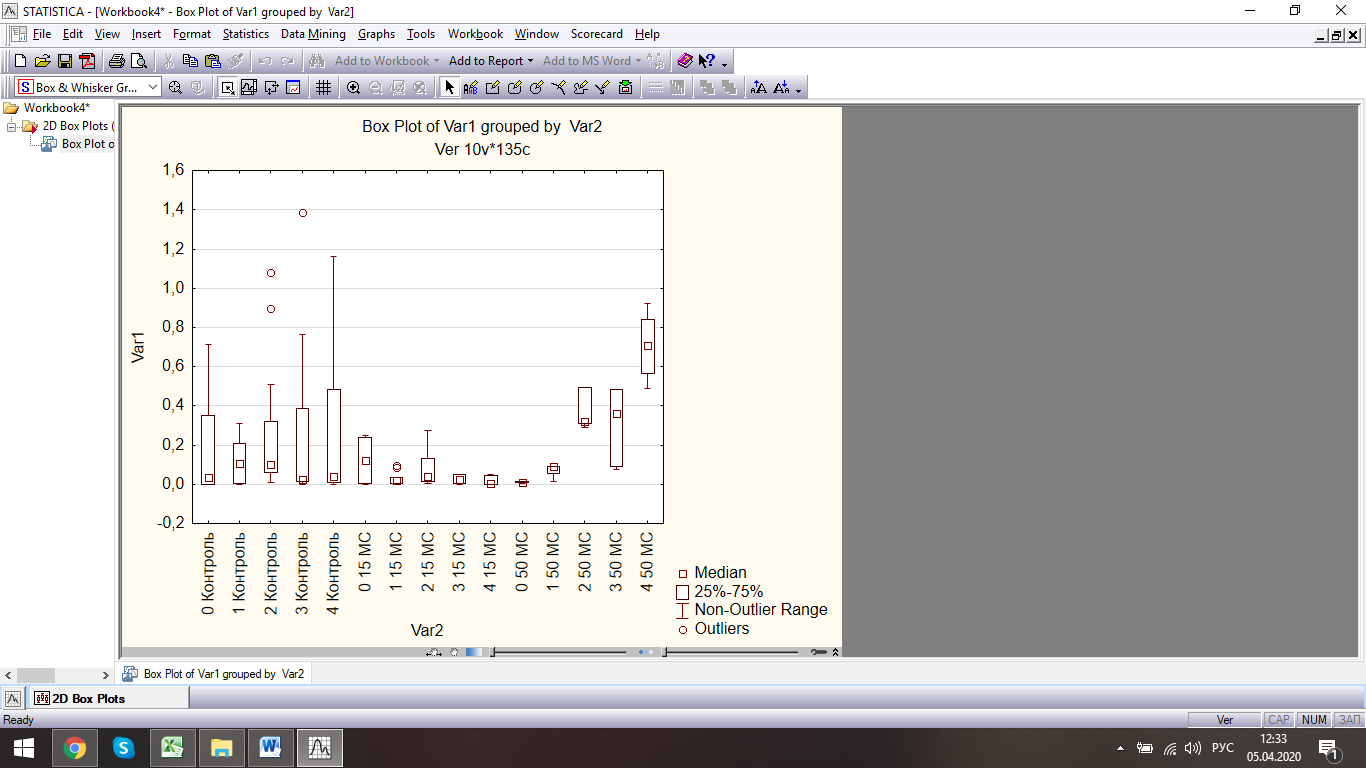

Supplement: S1 File — (DOCX) [file pone.0241784.s004.docx]
